# Carotid Intima‐Media Thickness but Not Carotid Artery Plaque in Healthy Individuals Is Linked to Lean Body Mass

**DOI:** 10.1161/JAHA.118.011919

**Published:** 2019-07-31

**Authors:** Matthew Arnold, Andrew Linden, Robert Clarke, Yu Guo, Huaidong Du, Zheng Bian, Eric Wan, Meng Yang, Liang Wang, Yuexin Chen, Jianwei Chen, Huajun Long, Qijun Gu, Rory Collins, Liming Li, Zhengming Chen, Sarah Parish, the China Kadoorie Biobank Collaborative Group, Junshi Chen, Jun Lv, Richard Peto, Robin Walters, Derrick Bennett, Ruth Boxall, Fiona Bragg, Yumei Chang, Yiping Chen, Simon Gilbert, Alex Hacker, Michael Holmes, Christiana Kartsonaki, Rene Kerosi, Garry Lancaster, Kuang Lin, John McDonnell, Iona Millwood, Qunhua Nie, Pang Yao, Paul Ryder, Sam Sansome, Dan Schmidt, Rajani Sohoni, Iain Turnbull, Robin Walters, Jenny Wang, Lin Wang, Neil Wright, Ling Yang, Xiaoming Yang, Xiao Han, Can Hou, Biao Jing, Chao Liu, Jun Lv, Pei Pei, Yunlong Tan, Canqing Yu, Ruqin Gao, Shanpeng Li, Shaojie Wang, Yongmei Liu, Ranran Du, Yajing Zang, Liang Cheng, Xiaocao Tian, Hua Zhang, Yaoming Zhai, Feng Ning, Xiaohui Sun, Feifei Li, Silu Lv, Junzheng Wang, Wei Hou, Mingyuan Zeng, Ge Jiang, Liqiu Yang, Hui He, Bo Yu, Yanjie Li, Qinai Xu, Quan Kang, Dan Wang, Ximin Hu, Hongmei Wang, Jinyan Chen, Yan Fu, Zhenwang Fu, Xiaohuan Wang, Min Weng, Zhendong Guo, Shukuan Wu, Yilei Li, Huimei Li, Zhifang Fu, Ming Wu, Yonglin Zhou, Jinyi Zhou, Ran Tao, Jie Yang, Jian Su, Fang Liu, Jun Zhang, Yihe Hu, Yan Lu, Liangcai Ma, Aiyu Tang, Shuo Zhang, Jianrong Jin, Jingchao Liu, Zhenzhu Tang, Naying Chen, Ying Huang, Mingqiang Li, Jinhuai Meng, Rong Pan, Qilian Jiang, Jian Lan, Yun Liu, Liuping Wei, Liyuan Zhou, Ningyu Chen, Ping Wang, Fanwen Meng, Yulu Qin, Sisi Wang, Xianping Wu, Ningmei Zhang, Xiaofang Chen, Weiwei Zhou, Guojin Luo, Jianguo Li, Xiaofang Chen, Xunfu Zhong, Jiaqiu Liu, Qiang Sun, Pengfei Ge, Xiaolan Ren, Caixia Dong, Hui Zhang, Enke Mao, Xiaoping Wang, Tao Wang, Xi Zhang, Ding Zhang, Gang Zhou, Shixian Feng, Liang Chang, Lei Fan, Yulian Gao, Tianyou He, Huarong Sun, Pan He, Chen Hu, Xukui Zhang, Huifang Wu, Pan He, Min Yu, Ruying Hu, Hao Wang, Yijian Qian, Chunmei Wang, Kaixu Xie, Lingli Chen, Yidan Zhang, Dongxia Pan, Yuelong Huang, Biyun Chen, Li Yin, Huilin Liu, Zhongxi Fu, Qiaohua Xu, Xin Xu, Hao Zhang, Xianzhi Li, Libo Zhang, Zhe Qiu

**Affiliations:** ^1^ Clinical Trial Service Unit and Epidemiological Studies Unit Nuffield Department of Population Health University of Oxford Oxford United Kingdom; ^2^ MRC Population Health Research Unit University of Oxford Oxford United Kingdom; ^3^ Chinese Academy of Medical Sciences Beijing China; ^4^ Division of Ultrasound Diagnosis Peking Union Medical College Hospital Beijing China; ^5^ Centre of Vascular Surgery Peking Union Medical College Hospital Beijing China; ^6^ Liuyang CDC Liuyang China; ^7^ NCDs Prevention and Control Department Liuyang CDC Liuyang China; ^8^ Tongxiang CDC Tongxiang China; ^9^ Department of Epidemiology and Biostatistics School of Public Health Peking University Health Science Center Beijing China

**Keywords:** atherosclerosis, carotid intima‐media thickness, lean body mass, Atherosclerosis, Primary Prevention, Ultrasound, Race and Ethnicity, Vascular Biology

## Abstract

**Background:**

Lean body mass has been identified as a key determinant of left ventricular mass and wall thickness. However, the importance of lean body mass or other body‐size measures as normative determinants of carotid intima‐media thickness (cIMT), a widely used early indicator of atherosclerosis, has not been well established.

**Methods and Results:**

Carotid artery ultrasound measurements of cIMT and carotid artery plaque burden (derived from plaque number and maximum size) and measurements of body size, including height, body mass index, weight, body fat proportion, and lean body mass ([1−body fat proportion]×weight), were recorded in 25 020 participants from 10 regions of China. Analyses were restricted to a healthy younger subset (n=6617) defined as never or long‐term ex‐regular smokers aged <60 years (mean age, 50) without previous ischemic heart disease, stroke, diabetes mellitus, or hypertension and with plasma non‐high‐density lipoprotein cholesterol <4 mmol/L. Among these 6617 participants, 86% were women (because most men smoked) and 9% had carotid artery plaque. In both women and men separately, lean body mass was strongly positively associated with cIMT, but was not associated with plaque burden: overall, each 10 kg higher lean body mass was associated with a 0.03 (95% CI, 0.03–0.04) mm higher cIMT (*P*=5×10^−33^). Fat mass, height, and other body‐size measures were more weakly associated with cIMT.

**Conclusions:**

The strong association of lean body mass with cIMT, but not with plaque burden, in healthy adults suggests a normative relationship rather than reflecting atherosclerotic pathology. Common mechanisms may underlie the associations of lean body mass with cIMT and with nonatherosclerotic vascular traits.

**Clinical Perspective**

**What Is New?**

- This is the first study to examine the association of body‐size measures with both carotid intima‐media thickness (cIMT) and carotid plaque in healthy individuals.
- Lean body mass was strongly positively associated with cIMT, but was not associated with carotid plaque burden, suggesting that the association reflected a normative relationship rather than atherosclerotic pathology.

**What Are the Clinical Implications?**

- In younger people, below the age of around 50 years, carotid plaque is likely to be rare and cIMT offers valuable early evidence of atherosclerotic change, but the normal relationship of body size to cIMT needs to be taken into account. The findings suggest that cIMT should be corrected for lean body mass.
- Given that lean body mass has also been identified as the leading body‐size measure associated with left ventricular mass and atrial fibrillation, there may be a common causal mechanism linking these associations, such as a link between lean body mass and artery and heart wall thicknesses.

## Introduction

Atherosclerosis is a major cause of morbidity and mortality globally, and there is a need to detect and treat it as early as possible.^1^ Carotid intima‐media thickness (cIMT) is a measure of hypertrophy of the wall of the carotid arteries involving thickening of the intima and media layers and is a widely used, noninvasive early indicator of subclinical vascular disease.^2^ Carotid plaque is a focal thickening of localized regions of the intimal layer of the arterial wall that may progress and cause stenosis of the affected artery or rupture and result in embolism to the brain.^2^, ^3^ The burden of carotid atherosclerosis increases strongly with age, and measures of carotid plaque are stronger predictors than cIMT of cardiovascular disease (CVD) in middle‐aged and older adults.^2^, ^4^, ^5^, ^6^ In children and young adults, carotid plaque is rare and cIMT is an important earlier marker of atherosclerotic risk in individuals with comorbidities associated with high risk of cardiovascular disease (CVD).^7^, ^8^, ^9^

Mean cIMT increases progressively from childhood until adulthood with increasing age, height, and body mass index (BMI) and varies by ethnicity.^7^, ^10^, ^11^, ^12^ In addition, recent studies of obese individuals have suggested that lean body mass is the strongest determinant of ethnic differences in cIMT.^13^, ^14^ Whether such associations reflect normal vascular adaptation or disease‐related changes is unclear, particularly in studies among older or less healthy individuals.^7^, ^10^ Lean body mass has also been identified as the chief body‐size risk factor for left ventricular mass and atrial fibrillation, raising the possibility of a mechanistic link.^15^, ^16^, ^17^, ^18^, ^19^

A systematic search for studies examining the relationship of cIMT to ethnicity, height, and lean body mass in younger, healthy populations^11^, ^13^, ^14^, ^20^, ^21^, ^22^, ^23^, ^24^, ^25^, ^26^, ^27^, ^28^, ^29^, ^30^, ^31^, ^32^, ^33^ (Data S1; Tables S1 and S2) found positive associations of height with cIMT in the 3 largest studies in young people (≈1700 children and adolescents).^14^, ^29^, ^30^, ^31^ No studies have investigated associations of lean body mass with cIMT in healthy children, but 1 small study of young adults and 2 studies of overweight individuals reported positive associations of lean body mass with cIMT.^13^, ^14^ There was strong evidence for variation in cIMT by ethnicity, with higher mean levels of cIMT in blacks than in whites. The 1 study also reporting on carotid artery plaque found that cIMT, but not plaque, was higher in blacks than whites, suggesting that ethnic differences in cIMT may not be indicators of early atherosclerosis.^34^ Further studies are needed to distinguish the determinants of normative values of cIMT from those of early atherosclerosis. The present report addresses this gap by examining the association of body‐size measures with both cIMT and carotid artery plaque in a large number of healthy individuals.

This report aims to (1) investigate the relationship of lean body mass and other body‐size measures to cIMT and carotid artery plaque in a healthy subset of 6617 participants aged <60 years from within a study of 25 020 adults recruited from 10 diverse and geographically distinct regions of China and (2) investigate whether lean body mass could account for regional variation in cIMT (as a proxy for variation attributable to ancestry).^35^

## Methods

Procedures for requesting the analytical methods and data tables that support the findings of this study are available on the Nuffield Department of Population Health website (https://www.ndph.ox.ac.uk/files/about/ndph-data-access-policy.pdf).^36^

### Study Population

Details of the design and methods of the China Kadoorie Biobank (CKB) have been previously reported.^37^ Overall, 512 891 adults aged 30 to 79 years were enrolled during 2004–2008 from 10 diverse regions in China. In the baseline survey, information was collected on demographic and socioeconomic status, lifestyle behaviors, and medical history using interviewer‐administered questionnaires. Ethical approval for the CKB Study was obtained from the Oxford Tropical Research Ethics Committee at the University of Oxford (OXTREC) and the Chinese Centre for Disease Control and Prevention (CDC) Ethical Review Committee. Ethical approval for the 2013–2014 resurvey was obtained from both OXTREC and the Chinese Academy of Medical Sciences and Peking Union Medical College Ethical Review committees. Approval for both the main study and the resurvey was also granted by the institutional boards at the CDCs in each of the survey sites. All participants provided written informed consent.

A resurvey of a random sample of 25 020 surviving participants conducted in 2013–2014 included a repeat of the baseline questionnaire and clinical measurements, plus additional clinical measurements not included at baseline. Measurements were recorded and blood samples collected in the nonfasting state. Body fat proportion was estimated using a bioelectrical impedance analyser (Data S1). Fat mass was calculated as body fat proportion multiplied by total body weight, and fat free mass (referred to as lean body mass in this report) was calculated as (1−body fat proportion) multiplied by total body weight.

Automated B‐mode ultrasound screening of the extracranial carotid arteries (by sonographers using a Panasonic Cardio‐Health Station) recorded measurements of the mean cIMT of the common carotid artery, the number of carotid plaques (defined as focal thickenings of intima‐media >1.5 mm, consistent with the Mannheim consensus)^2^ and preplaques (>1.0 and ≤1.5 mm), and thickness of the largest plaque. Repeat measurements by a radiologist in a subset yielded an overall quality score of 85%, reflecting the extent to which the radiologists agreed with the sonographers (as previously reported^38^). Plaque measurements were combined to form a plaque burden (in mm, interpretable as an enhanced estimate of the maximum plaque thickness), as previously described.^38^ Other measurements available included anthropometric and adiposity measures, blood pressure, blood total and high‐density lipoprotein (HDL) cholesterol concentrations, forced expiratory volume in 1 second, forced vital capacity, hand‐grip strength, arterial stiffness, and heel bone mineral density (Data S1).

The study population for the present report was defined as the 6617 participants with carotid measurements who were aged <60 years at resurvey and who had: no previous history of CVD (heart disease, stroke, or transient ischemic attack); no previous history of diabetes mellitus (reported diagnosis, detected from blood glucose at resurvey or identified from medical records during previous follow‐up); no reported diagnosis of hypertension and with systolic blood pressure (SBP) <160 mm Hg and diastolic blood pressure (DBP) <100 mm Hg; were not regular smokers at either baseline or resurvey and had not stopped smoking because of disease; and who had non‐HDL cholesterol at resurvey <4 mmol/L. Age <60 years was included as a criterion because age is a strong risk factor for atherosclerosis, even in the absence of the other risk factors. After exclusion of 49 participants missing body fat proportion or non‐HDL cholesterol and 6 participants with extreme BMI (<15 or >40 kg/m^2^) or extreme waist circumference (<50 or >125 cm), 6617 healthy younger participants remained as the analysis population. Older and unhealthy participants without previous CVD were included in a sensitivity analysis.

### Statistical Analysis

Analyses were based on measurements recorded at the resurvey in 2013–2014. For each of lean body mass, SBP, and non‐HDL cholesterol, 5 groups were defined by quintiles of their respective distributions within each sex. Residuals of DBP were computed after adjustment for SBP (denoted “DBP given SBP”), in order to indicate any additional predictive value of DBP over SBP. In results presented per SD of cIMT and plaque burden, SDs computed over healthy participants aged <60 years were used (irrespective of the subset presented).

All regression models were linear and, where appropriate, were adjusted for the interaction of 5‐year age group, sex and region, and cardiovascular risk factors (SBP and DBP, non‐HDL cholesterol, and smoker status [former versus never]). Percentage of variation in carotid measures explained by various adjustments (from the factors listed in Table S3) was assessed using ANOVA type 1 sums‐of‐squares analyses. Correlations of the mean lean body mass levels within regions (after adjustment for age and smoking status) with the adjusted mean levels of the carotid measures and cardiovascular risk factors within regions were estimated separately for each sex using Pearson correlation coefficients. An indication of the robustness of the correlations was provided by the range (minimum and maximum) of the correlations when 1 region at a time was omitted. All analyses used SAS (version 9.4; SAS Institute Inc., Cary, NC) or R software (version 3.4.2; R Foundation for Statistical Analysis, Vienna, Austria).

## Results

### Characteristics of the Study Population

In the analysis population of 6617 healthy participants aged <60 years, 86% were women (because most men were smokers), mean age was 50 years, 63% lived in rural areas, and mean BMI was 24 kg/m^2^ (Table 1). Mean cIMT was 0.60 (0.10) mm, 9% had carotid plaque, and mean carotid plaque burden was 0.22 (0.56) mm.

**Table 1:** Characteristics of the Study Population at Resurvey

Characteristic at Resurvey	Women	Men	Total
Demography			
N	5676	941	6617
Age, y			
<50	2971 (52%)	485 (52%)	3456 (52%)
≥50	2705 (48%)	456 (48%)	3161 (48%)
Mean age, y	49.7 (5.3)	49.8 (5.4)	49.7 (5.3)
Urban residence	2096 (37%)	346 (37%)	2442 (37%)
Rural residence	3580 (63%)	595 (63%)	4175 (63%)
Major cardiovascular disease risk factors			
Systolic blood pressure, mm Hg	124 (14.1)	127 (12.6)	124 (13.9)
Ex‐regular smoker	7 (0%)	208 (22%)	215 (3%)
Non‐HDL cholesterol, mmol/L	2.43 (0.76)	2.47 (0.72)	2.43 (0.75)
HDL cholesterol, mmol/L	1.44 (0.44)	1.24 (0.42)	1.41 (0.44)
Body‐size measures			
Height, cm	155 (5.8)	166 (6.6)	157 (7.1)
Weight, kg	57.4 (8.8)	67.3 (10.4)	58.8 (9.7)
Body mass index, kg/m²	23.9 (3.2)	24.3 (3.0)	23.9 (3.2)
Waist circumference, cm	81.6 (8.8)	86.3 (9.0)	82.3 (9.0)
Waist‐hip ratio	0.91 (0.08)	0.96 (0.08)	0.92 (0.08)
Body fat, %	29.3 (6.4)	18.8 (5.9)	27.8 (7.3)
Fat body mass, kg	17.3 (6.0)	13.1 (5.5)	16.7 (6.1)
Lean body mass, kg	40.2 (4.0)	54.2 (6.4)	42.2 (6.6)
Carotid measures			
cIMT, mm	0.59 (0.10)	0.63 (0.11)	0.60 (0.10)
Carotid plaque burden, mm	0.20 (0.54)	0.31 (0.65)	0.22 (0.56)
Plaque (>1.5 mm) present	490 (9%)	129 (14%)	619 (9%)

Data are mean (SD) or number (%) of participants. The 6617 participants included are never or long‐term ex‐regular smokers, aged <60 years, without previous ischemic heart disease, stroke, diabetes mellitus, or hypertension, having plasma non‐HDL cholesterol <4 mmol/L. cIMT indicates carotid intima‐media thickness; HDL, high‐density lipoprotein.

### Body‐Size Measures and Carotid Measures

**Figure 1:**
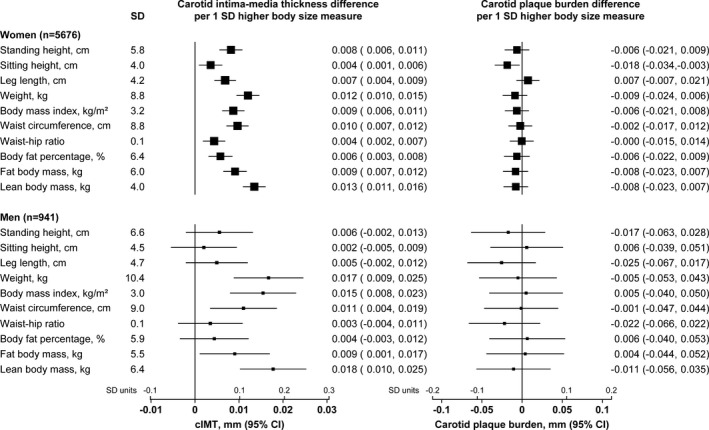
Sex‐specific associations of body‐size measures with carotid intima‐media thickness (cIMT) and carotid plaque burden. Associations are adjusted for age, systolic and diastolic blood pressure, non‐HDL cholesterol, smoking status and region. HDL indicates high‐density lipoprotein.

After adjustment for age, sex, region, and cardiovascular risk factors (SBP, DBP, non‐HDL cholesterol, and smoking status) most body‐size measures were strongly positively associated with cIMT, but showed no association with carotid plaque burden (Figure 1). Among the measures considered, lean body mass had the strongest positive association with cIMT in both women (0.013; 95% CI, 0.011–0.016; mm cIMT per SD lean body mass, *P*=8×10^−27^) and men (0.018; 0.010–0.025; *P*=2×10^−6^); weight had a similar strength of association, whereas other anthropometric measures, such as fat body mass, height, and BMI also had positive, but weaker, associations with cIMT (Figure 1). The associations of lean body mass and weight with cIMT remained strong after adjustment for each of the other body‐size measures individually (all remaining *P*<1×10^−12^ for lean body mass among the women; data not shown). By contrast, the associations of the other body‐size measures after adjustment for lean body mass were all *P*>0.01.

**Figure 2:**
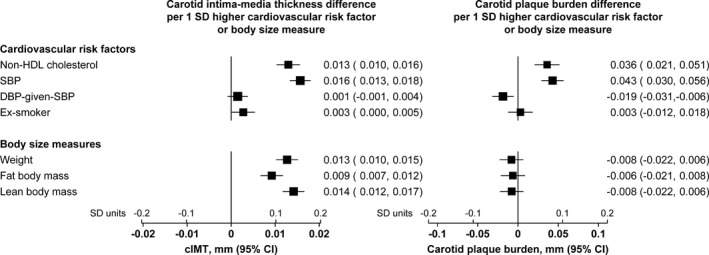
Concordant associations of cardiovascular risk factors with carotid intima‐media thickness (cIMT) and carotid plaque burden, but discordant associations of body‐size measures with cIMT and plaque burden. All associations are adjusted for age, sex, and region; associations of body‐size measures are additionally adjusted for the cardiovascular risk factors. The size of each square is proportional to the amount of statistical information. SDs of body‐size measures were computed separately in men and women. DBP indicates diastolic blood pressure; HDL, high‐density lipoprotein; SBP, systolic blood pressure.

This discordant pattern of association of the body‐size measures with the 2 carotid measures was in marked contrast with the pattern of association of the cardiovascular risk factors, such as SBP and non‐HDL cholesterol, which were positively associated with both of the carotid measures to a similar extent (Figure 2). This distinction between body‐size measures and cardiovascular risk factors in their associations with the 2 carotid measures was also apparent in a sensitivity analysis considering both healthy and unhealthy participants (Figure S1).

### Variation in Carotid Measures

Analysis of variance was conducted to identify all factors contributing to variation in the carotid measures in this healthy population selected to have low levels of cardiovascular risk factors. In the 5592 women with complete data on all covariates, age explained 15.5% of the variance in cIMT, major cardiovascular risk factors explained a further 2.8%, and region explained a further 6.2%. After adjustment for these factors, lean body mass explained 2% of the residual variance; other body‐size measures explained smaller amounts and were the only other factors explaining at least 0.5% of the residual variance (Table 2). The pattern in the 924 men was similar, with lean body explaining 2.3% of the residual variance and other body‐size measures explaining smaller amounts, but, in addition, arterial stiffness accounted for 1% of the variance. For carotid plaque burden, in women, age explained 4.7% of the variance, major cardiovascular risk factors explained a further 1.2%, and region explained a further 6.4%. In men, age explained 9.4% of the variance, major cardiovascular risk factors explained a further 0.7%, and region explained a further 8.3%. No other factors explained at least 0.5% of the residual variance of carotid plaque burden in either women or men.

**Table 2:** Percentage of Variation in Carotid Intima‐Media Thickness and Carotid Plaque Burden Explained by Factors

Adjustment	Carotid Intima‐media Thickness	Carotid Plaque Burden
	Percentage of Variation Explained	Percentage of Variation Explained
Women (n=5591)		
Age	15.5%	4.7%
+Major CVD risk factors	18.3%	5.9%
+Region	24.5%	12.3%
After adjustment for age, CVD risk factors, and region		
Lean body mass	2.0%	
Weight	1.6%	
Waist circumference	1.1%	
Fat mass	0.9%	
Body mass index	0.9%	
Standing height	0.7%	
Leg length	0.5%	
Men (n=924)		
Age	13.6%	9.4%
+Major CVD risk factors	17.6%	10.1%
+Region	23.5%	18.4%
After adjustment for age, CVD risk factors, and region		
Lean body mass	2.3%	
Weight	1.7%	
Body mass index	1.7%	
Arterial stiffness	1.0%	
Waist circumference	0.8%	

Percentages given in the age, major CVD risk factors, and region rows are of total variation in carotid measure; percentages given in other rows are of the remaining variation in the carotid measure after adjustment for age, major CVD risk factors, and region. The table includes variables that explain at least 0.5% of variation in carotid measure. The list of variables available for selection is given in Table S3. Eighty‐five of the 6617 participants were excluded because of missing covariates. CVD indicates cardiovascular disease.

### Quantitative Relationships Between Lean Body Mass and cIMT

**Figure 3:**
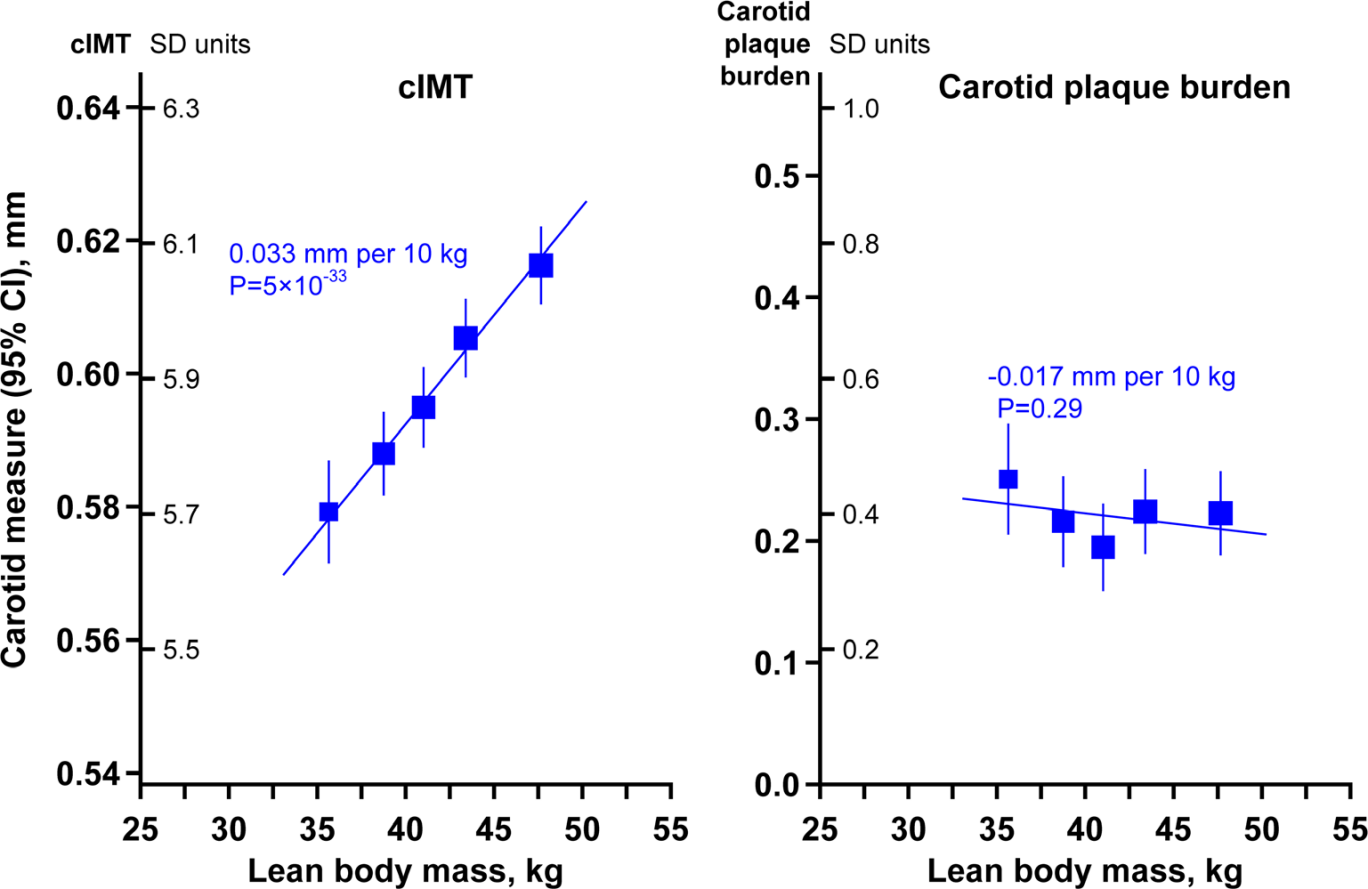
The associations of carotid intima‐media thickness (cIMT) and carotid plaque burden with lean body mass. Associations are adjusted for age, sex, region, systolic and diastolic blood pressure, non‐high‐density lipoprotein cholesterol, and smoking status.

**Figure 4:**
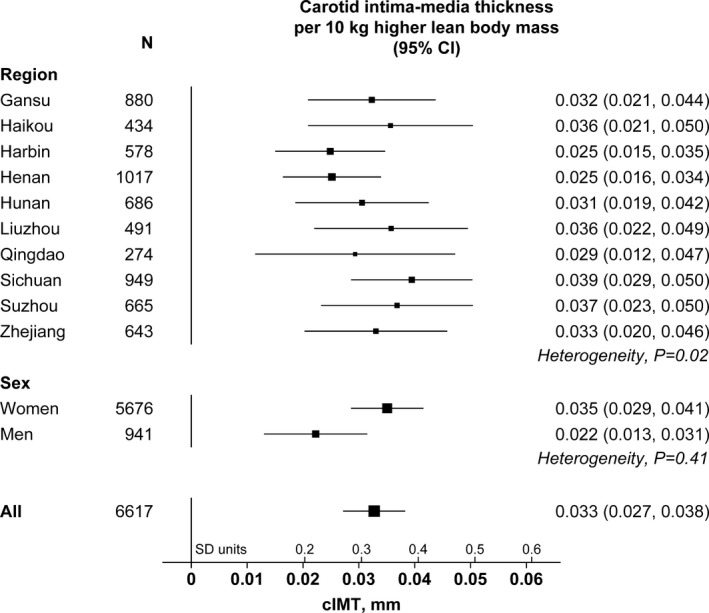
Carotid intima‐media thickness (cIMT) per 10 kg higher lean body mass by region and sex. Associations are adjusted for region and sex (as appropriate) and for age, systolic and diastolic blood pressure, non‐high‐density lipoprotein cholesterol, and smoking status. The size of each square is proportional to the amount of statistical information. SD of cIMT: 0.10 mm.

The associations of lean body mass with cIMT correspond to a trend of 0.033 (95% CI, 0.027–0.038) mm higher cIMT per 10 kg higher lean body mass (*P*=5×10^−33^) and a lack of statistically significant association with plaque burden (Figure 3). There was no heterogeneity in the association with cIMT by region or sex (Figure 4).

### Regional Variation in Carotid Measures

Region accounted for 6% of the variation in cIMT in both women and men and also for 6% and 8% of the variation in carotid plaque burden in women and men, respectively (Table 2). To investigate whether these percentages of variance could be attributable to the differences in lean body mass across regions, the means of lean body mass, carotid measures, and cardiovascular risk factors within each region are shown in Table 3. There was a strong positive correlation (*r*=0.79) between mean lean body mass and mean cIMT across regions in the 5676 women, which was much stronger than the correlation between mean lean body mass and mean plaque burden across regions (*r*=0.34). These correlations were robust when 1 region at a time was omitted (Table 3). In the 941 men, the correlation of mean lean body mass with mean cIMT was also positive, but weaker (*r*=0.40), and more similar to the correlation with carotid plaque burden (*r*=0.27). Correlation coefficients between the mean lean body mass in regions and the means of non‐HDL cholesterol and SBP were also around this absolute level. The number of regions may be insufficient to draw an interpretation from the moderate correlations in the more‐limited number of men.

**Table 3:** Correlations of the Mean Lean Body Mass Within Regions With the Mean Levels of Carotid Measures and Risk Factors Within the Regions

Region	N	Lean Body Mass, kg	cIMT, mm	Carotid Plaque Burden, mm	Non‐HDL Cholesterol, mmol/L	Systolic BP, mm Hg
Women	5676					
Suzhou	600	37.9 (0.2)	0.578 (0.004)	0.10 (0.02)	2.67 (0.03)	127.9 (0.6)
Liuzhou	418	39.2 (0.2)	0.591 (0.004)	0.40 (0.03)	3.02 (0.03)	123.6 (0.7)
Hunan	584	39.2 (0.2)	0.601 (0.004)	0.06 (0.02)	2.80 (0.03)	125.1 (0.6)
Zhejiang	568	39.5 (0.2)	0.573 (0.004)	0.09 (0.02)	2.28 (0.03)	123.7 (0.6)
Sichuan	833	39.6 (0.1)	0.558 (0.003)	0.08 (0.02)	2.37 (0.02)	121.1 (0.5)
Haikou	383	40.5 (0.2)	0.612 (0.005)	0.38 (0.03)	2.38 (0.03)	123.2 (0.7)
Henan	803	40.9 (0.1)	0.598 (0.003)	0.32 (0.02)	2.49 (0.02)	124.1 (0.5)
Gansu	792	41.2 (0.1)	0.613 (0.003)	0.20 (0.02)	1.66 (0.02)	121.9 (0.5)
Harbin	451	41.9 (0.2)	0.622 (0.004)	0.36 (0.02)	2.82 (0.03)	126.2 (0.6)
Qingdao	244	43.8 (0.2)	0.633 (0.006)	0.20 (0.03)	2.06 (0.04)	122.5 (0.9)
Correlation with lean body mass (range)*			0.79 (0.70, 0.88)	0.34 (0.23, 0.54)	−0.46 (−0.63, −0.32)	−0.38 (−0.58, −0.03)
Men	941					
Sichuan	116	51.6 (0.5)	0.601 (0.009)	0.13 (0.06)	2.34 (0.06)	122.2 (1.1)
Hunan	102	51.6 (0.6)	0.644 (0.010)	0.11 (0.06)	2.68 (0.06)	130.0 (1.2)
Suzhou	65	51.7 (0.7)	0.625 (0.013)	0.16 (0.08)	2.55 (0.08)	131.7 (1.5)
Liuzhou	73	52.1 (0.7)	0.651 (0.012)	0.51 (0.07)	3.02 (0.07)	127.1 (1.4)
Zhejiang	75	53.5 (0.7)	0.615 (0.012)	0.19 (0.07)	2.24 (0.07)	123.9 (1.4)
Haikou	51	53.8 (0.8)	0.658 (0.014)	0.41 (0.09)	2.58 (0.09)	124.5 (1.7)
Gansu	88	54.1 (0.6)	0.664 (0.011)	0.46 (0.07)	1.67 (0.07)	125.2 (1.3)
Henan	214	55.4 (0.4)	0.616 (0.007)	0.37 (0.04)	2.43 (0.04)	126.7 (0.8)
Harbin	127	58.5 (0.5)	0.655 (0.009)	0.46 (0.05)	2.89 (0.06)	133.1 (1.1)
Qingdao	30	59.7 (1.1)	0.654 (0.019)	0.19 (0.11)	1.85 (0.12)	129.4 (2.2)
Correlation with lean body mass (range)*			0.40 (0.27, 0.49)	0.27 (0.09, 0.59)	−0.27 (−0.60, 0.07)	0.38 (0.12, 0.59)

Regions are ordered by the mean lean body mass within women and men. BP indicates blood pressure; cIMT, carotid intima‐media thickness; HDL, high‐density lipoprotein.

* Pearson correlation coefficients between the lean body mass and other factor means and the range of correlation coefficients when 1 region is omitted.

## Discussion

In healthy middle‐aged adults aged <60 years, lean body mass was strongly positively associated with cIMT, but was not associated with carotid plaque burden. This suggests that the association of lean body mass with cIMT reflects a normative relationship rather than atherosclerotic pathology. Each 10 kg higher lean body mass was associated with a 0.03 (95% CI, 0.03–0.04) mm higher cIMT (*P*=5×10^−33^), after adjustment for age, sex, region, blood pressure, non‐HDL cholesterol, and former versus never smoking.

This is the first study to examine the association of body‐size measures with both cIMT and carotid plaque in healthy individuals. The findings in the present study of 6617 healthy Chinese adults (5676 women and 941 men) are supported by results in much smaller studies in overweight individuals. In a study of 421 overweight middle‐aged European men and women, a 10 kg higher lean body mass was associated with approximately 0.05 mm higher cIMT, but was not associated with carotid plaque.^13^ In the present study, other body‐size and adiposity measures were also associated with cIMT, but not with carotid plaque burden, showing that associations of adiposity with cIMT should not be interpreted as reflecting associations of adiposity with atherosclerosis.

Across the 10 ethnically diverse regions of China in the present study, in women there was a strong association of mean lean body mass with mean cIMT. This was not robustly confirmed in men, possibly because of inadequate data. The observations in women are consistent with a previous report in 120 overweight boys and girls in the United States, which found that variation in cIMT with black versus white ethnicity was accounted for by differences in lean body mass.^14^ Associations in less‐healthy populations may be confounded by greater atherosclerotic effects of cardiovascular risk factors and reverse causal effects of smoking (such as weight loss) and so provide less‐robust estimates of the relationship (as suggested by the results in the unhealthy or older participants in the present study; Figure S1).

Height was also associated with higher cIMT and not with greater carotid plaque burden, but the association with cIMT was weaker than that for lean body mass and was largely attenuated by adjustment for lean body mass. In this population of healthy adults, height accounted for only 0.7% of the variation in cIMT in women (and <0.5% in men), whereas lean body mass accounted for around 2% in both women and men (Table 2). The findings suggest that setting reference levels for cIMT according to lean body mass may be more appropriate than setting them according to height, but the quantitative relationships of lean body mass and height to cIMT require direct confirmation in healthy boys and girls at varying stages of growth, among whom lean body mass and height would be expected to explain a much greater proportion of the variation in cIMT than in the present population.

The present results parallel findings that lean body mass is the most relevant measure for determining reference levels of left ventricular mass in children, for which various lines of evidence likewise support the interpretation that the association reflects a normal adaptive response rather than a pathological effect.^15^, ^16^, ^39^ For example, when lean body mass increases through physical training, left ventricular mass and wall thickness also increase.^19^, ^40^ Height was also more weakly associated than lean body mass with left ventricular mass. A further study reported that in obesity, lean body mass appeared to be the main driver behind total left ventricular mass increase.^41^ The physical effort of carrying around extra body fat has been suggested to act like physical training in its effect on lean body mass.^19^

Lean body mass has also been identified as the chief anthropometric determinant of atrial fibrillation (stronger than the well‐established risk factors, height and BMI).^18^ Furthermore, Mendelian randomization analyses have shown that both lean body mass and height‐associated genetic variants are also associated with atrial fibrillation, suggesting that their relationships to atrial fibrillation are causal.^42^, ^43^ There may be a common causal mechanism linking lean body mass to higher cIMT, higher left ventricular mass, and greater risk of atrial fibrillation, such as a link between lean body mass and arterial and cardiac wall thickness. Thus, although thickening of cIMT attributable to lean body mass does not appear to reflect the beginning of atherosclerosis, whether it is adversely associated with any nonatherosclerotic diseases remains unclear.

Evidence on whether changes to cIMT reflecting growth and healthy increases in lean body mass differ from those induced by cardiovascular risk factors, such as smoking, lipids, and blood pressure, is very limited. In a study among 70‐year‐olds that recorded separate measurements of the thicknesses of the carotid intima and media layers noninvasively, CVD was associated with a thicker intima, but thinner media, layer.^44^ BMI was associated with a thicker intima layer, but lean body mass was not measured in this population. It would be valuable to assess the relationship of lean body mass to the thicknesses of the 2 layers in young adults. It might be hypothesized that healthy changes would affect the intima and media to a similar extent—or even increase the smooth muscle media layer to a greater extent. Previous studies have reported complex relationships between body dimensions and carotid artery dimensions.^45^, ^46^ Dimensional measurements (such as distances and volumes) from imaging studies often have strong correlations with body‐size measures.^47^, ^48^

### Limitations

A limitation of the present study was that it only included adults, and the quantitative relationships of lean body mass and height to cIMT require direct confirmation in healthy boys and girls at varying stages of growth. However, it is unlikely to be feasible to reliably replicate the lack of association with carotid plaque in such populations because the prevalence of carotid plaque depends strongly on age and is likely to be too low in children. (Among healthy individuals aged 40 to 45 years in the present study, only 3% had at least 1 carotid plaque >1.5 mm.) Hence, extrapolation from older populations, as in the present analysis, is a valuable approach. A further limitation of the present study was that the healthy adults were predominantly women.

## Appendix

Members of the China Kadoorie Biobank Collaborative Group *International Steering Committee:* Junshi Chen, Zhengming Chen (PI), Robert Clarke, Rory Collins, Yu Guo, Liming Li (PI), Jun Lv, Richard Peto, and Robin Walters. *International Co‐ordinating Centre, Oxford:* Daniel Avery, Derrick Bennett, Ruth Boxall, Fiona Bragg, Yumei Chang, Yiping Chen, Zhengming Chen, Robert Clarke, Huaidong Du, Simon Gilbert, Alex Hacker, Michael Holmes, Christiana Kartsonaki, Rene Kerosi, Garry Lancaster, Kuang Lin, John McDonnell, Iona Millwood, Qunhua Nie, Pang Yao, Paul Ryder, Sam Sansome, Dan Schmidt, Rajani Sohoni, Iain Turnbull, Robin Walters, Jenny Wang, Lin Wang, Neil Wright, Ling Yang, and Xiaoming Yang. *National Co‐ordinating Centre, Beijing:* Zheng Bian, Yu Guo, Xiao Han, Can Hou, Biao Jing, Chao Liu, Jun Lv, Pei Pei, Yunlong Tan, and Canqing Yu. *Regional Co‐ordinating Centres:* Qingdao CDC: Zengchang Pang, Ruqin Gao, Shanpeng Li, Shaojie Wang, Yongmei Liu, Ranran Du, Yajing Zang, Liang Cheng, Xiaocao Tian, Hua Zhang, Yaoming Zhai, Feng Ning, Xiaohui Sun, Feifei Li. Licang CDC: Silu Lv, Junzheng Wang, Wei Hou. Heilongjiang Provincial CDC: Mingyuan Zeng, Ge Jiang, Xue Zhou. Nangang CDC: Liqiu Yang, Hui He, Bo Yu, Yanjie Li, Qinai Xu, Quan Kang, Ziyan Guo. Hainan Provincial CDC: Dan Wang, Ximin Hu, Hongmei Wang, Jinyan Chen, Yan Fu, Zhenwang Fu, Xiaohuan Wang. Meilan CDC: Min Weng, Zhendong Guo, Shukuan Wu, Yilei Li, Huimei Li, Zhifang Fu. Jiangsu Provincial CDC: Ming Wu, Yonglin Zhou, Jinyi Zhou, Ran Tao, Jie Yang, Jian Su. Suzhou CDC: Fang Liu, Jun Zhang, Yihe Hu, Yan Lu, Liangcai Ma, Aiyu Tang, Shuo Zhang, Jianrong Jin, Jingchao Liu. Guangxi Provincial CDC: Zhenzhu Tang, Naying Chen, Ying Huang. Liuzhou CDC: Mingqiang Li, Jinhuai Meng, Rong Pan, Qilian Jiang, Jian Lan, Yun Liu, Liuping Wei, Liyuan Zhou, Ningyu Chen, Ping Wang, Fanwen Meng, Yulu Qin, Sisi Wang. Sichuan Provincial CDC: Xianping Wu, Ningmei Zhang, Xiaofang Chen, Weiwei Zhou. Pengzhou CDC: Guojin Luo, Jianguo Li, Xiaofang Chen, Xunfu Zhong, Jiaqiu Liu, Qiang Sun. Gansu Provincial CDC: Pengfei Ge, Xiaolan Ren, Caixia Dong. Maiji CDC: Hui Zhang, Enke Mao, Xiaoping Wang, Tao Wang, Xi Zhang. Henan Provincial CDC: Ding Zhang, Gang Zhou, Shixian Feng, Liang Chang, Lei Fan. Huixian CDC: Yulian Gao, Tianyou He, Huarong Sun, Pan He, Chen Hu, Xukui Zhang, Huifang Wu, Pan He. Zhejiang Provincial CDC: Min Yu, Ruying Hu, Hao Wang. Tongxiang CDC: Yijian Qian, Chunmei Wang, Kaixu Xie, Lingli Chen, Yidan Zhang, Dongxia Pan, Qijun Gu. Hunan Provincial CDC: Yuelong Huang, Biyun Chen, Li Yin, Huilin Liu, Zhongxi Fu, Qiaohua Xu. Liuyang CDC: Xin Xu, Hao Zhang, Huajun Long, Xianzhi Li, Libo Zhang, and Zhe Qiu.

## Sources of Funding

This work was supported by the UK Medical Research Council (MRC_MC_U137686851, MRC_MC_U137686853); the British Heart Foundation (CH/1996001/9454); Cancer Research UK (C500/A16896); the Kadoorie Charitable Foundation (during 2002–2009); the Wellcome Trust (104085/Z/14/Z); and the National Natural Science Foundation of China (81390541).

## Disclosures

Dr Collins reported support from the Nuffield Department of Population Health (drawing on expertise developed during research funded by both commercial and academic funders) during the conduct of the study and grants and personal fees from the British Heart Foundation, grants from Cancer Research UK, the Medical Research Council, Merck & Co, Wellcome Trust, and Medco, personal fees from UK Biobank, and a prize from Pfizer outside the conduct of the study; in addition, Dr Collins has a patent for a statin‐related myopathy genetic test issued and licensed. The authors declared no other potential conflicts of interest with respect to the research, authorship, and/or publication of this article.

## Supporting information


**Data S1.** Supplemental methods.
**Table S1.** Summary of Published Studies of the Associations of Carotid Intima‐Media Thickness (cIMT) With Ethnicity in Younger Individuals
**Table S2.** Summary of Published Studies of the Associations of Carotid Intima‐Media Thickness (cIMT) With Taller Height and Greater Lean Body Mass
**Table S3.** List of Variables Available for Selection in Analyses of Variation in Carotid Measures
**Figure S1.** Sensitivity analysis including healthy and unhealthy participants, showing concordant associations of cardiovascular risk factors with carotid intima‐media thickness (cIMT) and carotid plaque burden, but discordant associations of body‐size measures with cIMT and carotid plaque burden.
