# Supplementary material for: Carotid Intima‐Media Thickness but Not Carotid Artery Plaque in Healthy Individuals Is Linked to Lean Body Mass
Source: J Am Heart Assoc. 2019 Jul 31;8(15):e011919. doi: 10.1161/JAHA.118.011919 (PMC6761650; doi:10.1161/JAHA.118.011919)
Supplement: Supplementary file 1 — Data S1. Supplemental methods. Table S1. Summary of Published Studies of the Associations of Carotid Intima‐Media Thickness (cIMT) With Ethnicity in Younger Individuals Table S2. Summary of Published Studies of the Associations of Carotid Intima‐Media Thickness (cIMT) With Taller Height and Greater Lean Body Mass Table S3. List of Variables Available for Selection in Analyses of Variation in Carotid Measures Figure S1. Sensitivity analysis including healthy and unhealthy participants, showing concordant associations of cardiovascular risk factors with carotid intima‐media thickness (cIMT) and carotid plaque burden, but discordant associations of body‐size measures with cIMT and carotid plaque burden. [file JAH3-8-e011919-s001.pdf]

SUPPLEMENTAL MATERIAL

Data S1.

SUPPLEMENTAL METHODS

Evidence before this study

Publications that examined the relationships of cIMT with ethnicity, height, and lean body mass were identified using a systematic search of the PubMed and Embase databases on 31 August 2017. The following search terms (free text [searched in title or abstract] and relevant MeSH terms [PubMed] or subject headings [Embase]) and operators were used: cIMT (free text: carotid intim*, carotid atherosclerosis, cIMT, IMT, arterial thickness, arterial wall thickness) AND (ethnicity OR height OR lean body mass) [free text: ethni*, race, racial, ancestry, African, black, Asian, Chinese, Japanese, height, lean bod*, lean mass, fat free mass, muscle mass]. Articles were restricted to full length articles in peer reviewed English language journals and excluded if the study population was old (mean age ≥50 years) or had prior cardiovascular disease (obesity was permitted). After duplicates were removed, 3754 articles were screened by title and abstract and 49 by full text; the final synthesis included 17 articles (Tables S1 and S2).[1-17]

Strong evidence for variation in carotid intima-media thickness (cIMT) by ethnicity was found within the 11 studies (involving over 10 000 participants in total) investigating this (Table S1).[1-11] Compared to white participants, black participants had higher cIMT and this finding was consistent in studies of children and adults. Two studies in the US found that Hispanic and Asian (Korean and Japanese) participants had lower cIMT compared to white non-Hispanics, although a study of UK children saw no difference between white and Asian participants.

A small number of studies (with a total of about 3000 participants) examined the associations of height or lean body mass with cIMT in young populations (Table S2).[8, 12-17] In children and adolescents, taller height was associated with increased cIMT, but no similar association was seen in Korean adults. Increased lean body mass was associated with greater cIMT in children and adults with very high BMI and in a small study of Korean adolescent males.

Further details of study measurements

Smoking (in cigarettes per day), alcohol consumption (in grams per day), physical activity (in metabolic equivalent task hours) and sedentary time (in hours) were calculated from answers on the interview-administered questionnaire, as previously described.[18, 19] Body fat proportion (BFP) was estimated using a Tanita BC-418MA analyser using tetrapolar bioelectrical impedance and proprietary algorithms.[20] Non-fasting blood total cholesterol and high density lipoprotein (HDL) cholesterol were measured using a portable station (Mission Cholesterol Monitoring System). Forced Expiratory Volume in one second (FEV1) and Forced Vital Capacity (FVC) were measured by spirometry and the ratio FEV1/FVC calculated, as previously described.[21] Grip strength in each hand was measured using a dynamometer and the average used. Heel bone mineral density (in g/cm^2) was estimated from ultrasound measurements using the Sahara Clinical Bone Sonometer. Arterial stiffness was measured using a CareFusion Pulsetrace PCA 2 instrument and finger probe which measured the time taken for a pulse wave to travel through the arterial system and derived a stiffness index based on this and the person’s height.

Table S1. Summary of published studies of the associations of carotid intima-media thickness (cIMT) with ethnicity in younger individuals. Studies are grouped by the age or mean age of participants, to allow focus on studies in younger participants, as these are more likely to reflect normative associations rather than associations through disease, ageing and risk factors.

<table>
<tr><th rowspan="3">Author, year</th><th rowspan="3">Population</th><th rowspan="3">Study size</th><th rowspan="3">Mean age (SD) and range where available, years</th><th colspan="5">Association of cIMT with ethnicity*</th></tr>
<tr><th colspan="2">White</th><th>Black</th><th colspan="2">Asian</th></tr>
<tr><th>Non-Hispanic</th><th>Hispanic</th><th></th><th>Korean</th><th>Japanese</th></tr>
<tr><td colspan="9">Ethnicity (articles where all participants were under 50 years of age):</td></tr>
<tr><td>Tzou, 2007</td><td>US adults</td><td>1203</td><td>36 (4)
25-40</td><td colspan="2">Ref</td><td>↑</td><td colspan="2"></td></tr>
<tr><td>Whincup, 2012</td><td>UK children</td><td>939</td><td>10.8 (0.4)</td><td colspan="2">Ref</td><td>↑</td><td colspan="2">↔</td></tr>
<tr><td>Li, 2007</td><td>US adults</td><td>868</td><td>36.0 (4.4)
25-44†</td><td colspan="2">Ref</td><td>↑</td><td colspan="2"></td></tr>
<tr><td>Gao, 2016</td><td>US children and adults</td><td>784</td><td>18.0 (3.3)
10-24</td><td colspan="2">Ref</td><td>↑</td><td colspan="2"></td></tr>
<tr><td>Breton, 2011</td><td>US adults</td><td>768</td><td>19
18-25</td><td>Ref</td><td>↓</td><td>↑</td><td colspan="2">↓</td></tr>
<tr><td>Hao, 2016</td><td>US children and adults</td><td>521</td><td>24 (3.2)
13-36</td><td colspan="2">Ref</td><td>↑</td><td colspan="2"></td></tr>
<tr><td>Choo, 2008</td><td>Korean and Japanese men</td><td>352</td><td>45.1 (2.8)
40-49†</td><td colspan="2"></td><td></td><td>↑</td><td>Ref</td></tr>
<tr><td>Chowdhury, 2014</td><td>Europeans (BMI > 95th percentile)</td><td>120</td><td>12.1 (3.4)
4-21†</td><td colspan="2">Ref</td><td>↑</td><td colspan="2"></td></tr>
<tr><td colspan="9">Ethnicity (articles where the mean age of participants was <50 years):</td></tr>
<tr><td>Wendell, 2017</td><td>US adults</td><td>2270</td><td>47.7 (9.3)
30-64</td><td colspan="2">Ref</td><td>↑</td><td colspan="2"></td></tr>
<tr><td>Adolphe, 2009</td><td>US children and adults</td><td>2268</td><td>42.0 (11.4)
14-79</td><td>Ref</td><td>↓</td><td>↔</td><td colspan="2">↓</td></tr>
<tr><td>Hamer, 2011</td><td>South African adults</td><td>398</td><td>44.6 (9.7)
25-60†</td><td colspan="2">Ref</td><td>↑</td><td colspan="2"></td></tr>
</table>

* Ref Reference group, ↑ positive association with cIMT, ↓ inverse association with cIMT, ↔ non-significant association with cIMT. †Mean and SD age were calculated from values provided in the article.

Table S2. Summary of published studies of the associations of carotid intima-media thickness (cIMT) with taller height and greater lean body mass. Studies are grouped by the age or mean age of participants, to allow focus on studies in younger participants as these are more likely to reflect normative associations rather than associations through disease, ageing and risk factors.

Author, year	Population	Study size	Mean age (SD) and range where available, years	Association of cIMT with:* Taller height	Association of cIMT with:* Greater lean body mass
**Height (articles where all participants were under 50 years of age):**					
Doyon, 2013	European and Turkish children and adolescents	1155	6-18	↑	
Jourdan, 2005	German and Polish healthy adolescents	247	10-20	↑	
Finken, 2006	Dutch adolescents (born very preterm)	183	19 (0)<br>19-19	↑	
Chowdhury, 2014	Europeans (BMI > 95th percentile)	120	12.1 (3.4)<br>4-21†	↔	
**Height (articles where the mean age of participants was <50 years):**					
Song, 2011	Korean adults	706	45.0 (9.1)<br>30-74†	↔	
**Lean body mass (articles where all participants were under 50 years of age):**					
Chowdhury, 2014	Europeans (BMI > 95th percentile)	120	12.1 (3.4)<br>4-21†		↑
Kim, 2011	Korean adolescent males	92	17.0 (0.2)		↑
**Lean body mass (articles where the mean age of participants was <50 years):**					
Moreno, 2015	US adults (86% with BMI > 30 kg/m^2)	421	44.6 (9.8)<br>30-65†		↑

↑ positive association with cIMT, ↓ inverse association with cIMT, ↔ non-significant association with cIMT. †Mean and SD age were estimated from values provided in the article.

Table S3. List of variables available for selection in analyses of variation in carotid measures.

Variables available for selection	
Alcohol consumption (0)	Leg length (1)
Arterial stiffness (0)	Physical activity (met hours) (3)
Body fat percentage (0)	Sedentary time (3)
Body mass index (0)	Sitting height (1)
Bone mineral density (22)	Standing height (0)
FEV1 (74)	Waist circumference (0)
FEV1/FVC ratio (74)	Waist-hip ratio (0)
FVC (74)	Weight (0)
Fat mass (0)	
HDL cholesterol (0)	
Hand grip strength (3)	
Lean body mass (0)	

FEV1=forced expiry volume in 1 minute. FVC=forced vital capacity.

The number of participants missing a covariate (from the total of 6617 participants) is shown in brackets.

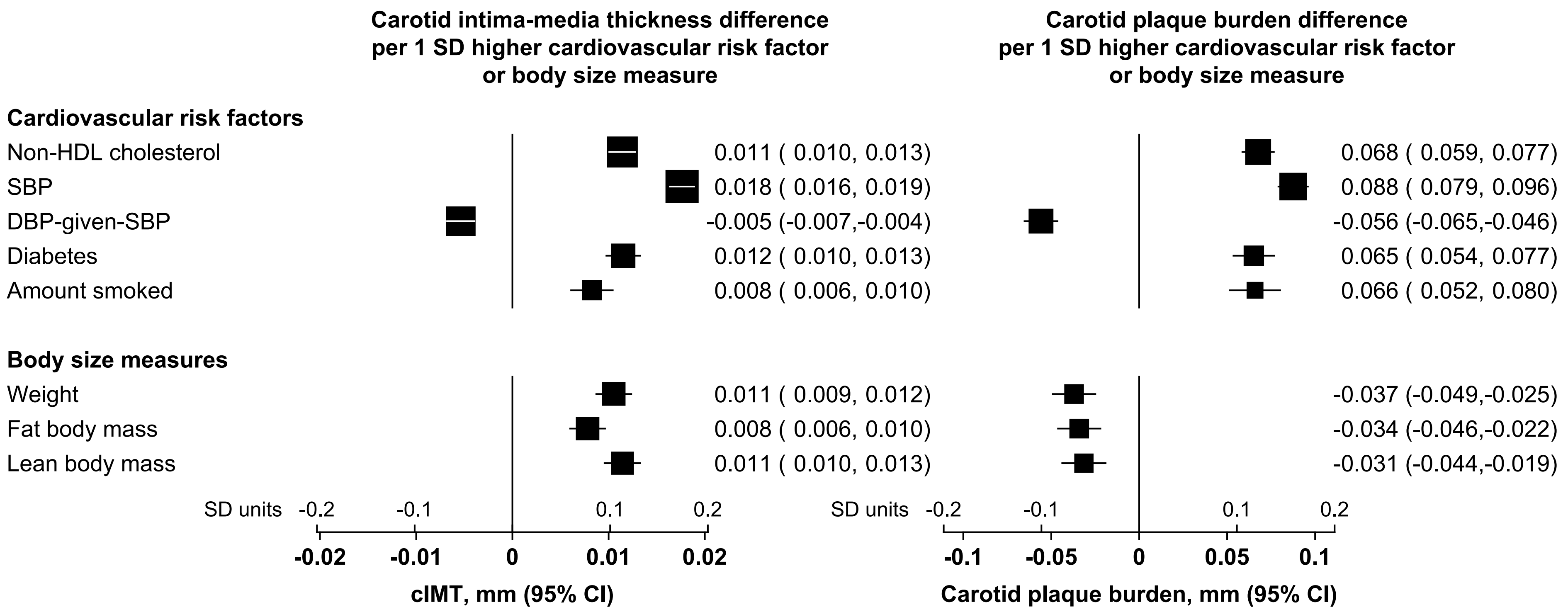

Figure S1. Sensitivity analysis including healthy and unhealthy participants, showing concordant associations of cardiovascular risk factors with carotid intima-media thickness (cIMT) and carotid plaque burden but discordant associations of body size measures with cIMT and carotid plaque burden. Analyses include 22 504 participants without prior cardiovascular disease. All associations are adjusted for age, sex and region; associations of body size measures are additionally adjusted for the cardiovascular risk factors. The size of each square is proportional to the amount of statistical information. For consistency, standard deviations (SDs) are as used in the main figures (i.e., computed in the healthy participants aged <60 years).

SUPPLEMENTAL REFERENCES:

1. Tzou WS, Douglas PS, Srinivasan SR, Bond MG, Tang R, Li S, Chen W, Berenson GS, Stein JH. Distribution and predictors of carotid intima-media thickness in young adults. Prev Cardiol. 2007; **10**: 181-9.
2. Whincup PH, Nightingale CM, Owen CG, Rapala A, Bhowruth DJ, Prescott MH, Ellins EA, Donin AS, Masi S, Rudnicka AR, Sattar N, Cook DG, Deanfield JE. Ethnic Differences in Carotid Intima-Media Thickness Between UK Children of Black African-Caribbean and White European Origin. Stroke. 2012; **43**: 1747-54.
3. Li S, Chen W, Srinivasan SR, Tang R, Bond MG, Berenson GS. Race (black-white) and gender divergences in the relationship of childhood cardiovascular risk factors to carotid artery intima-media thickness in adulthood: the Bogalusa Heart Study. Atherosclerosis. 2007; **194**: 421-5.
4. Gao Z, Khoury PR, McCoy CE, Shah AS, Kimball TR, Dolan LM, Urbina EM. Adiposity has no direct effect on carotid intima-media thickness in adolescents and young adults: Use of structural equation modeling to elucidate indirect & direct pathways. Atherosclerosis. 2016; **246**: 29-35.
5. Breton CV, Wang X, Mack WJ, Berhane K, Lopez M, Islam TS, Feng M, Hodis HN, Kunzli N, Avol E. Carotid artery intima-media thickness in college students: race/ethnicity matters. Atherosclerosis. 2011; **217**: 441-6.
6. Hao G, Wang X, Treiber FA, Davis H, Leverett S, Su S, Kapuku G. Growth of Carotid Intima-Media Thickness in Black and White Young Adults. J Am Heart Assoc. 2016; **5**: e004147.
7. Choo JN, Ueshima H, Jang Y, Sutton-Tyrrell K, El-Saed A, Kadowaki T, Takamiya T, Okamura T, Ueno Y, Nakamura Y, Sekikawa A, Curb JD, Kuller LH,

Shin C. Difference in carotid intima-media thickness between Korean and Japanese men. Annals of Epidemiology. 2008; **18**: 310-5.

8. Chowdhury SM, Henshaw MH, Friedman B, Saul JP, Shirali GS, Carter J, Levitan BM, Hulsey T. Lean Body Mass May Explain Apparent Racial Differences in Carotid Intima-Media Thickness in Obese Children. Journal of the American Society of Echocardiography. 2014; **27**: 561-7.
9. Wendell CR, Waldstein SR, Evans MK, Zonderman AB. Distributions of Subclinical Cardiovascular Disease in a Socioeconomically and Racially Diverse Sample. Stroke. 2017; **48**: 850-6.
10. Adolphe A, Cook LS, Huang X. A cross-sectional study of intima-media thickness, ethnicity, metabolic syndrome, and cardiovascular risk in 2268 study participants. Mayo Clin Proc. 2009; **84**: 221-8.
11. Hamer M, Malan L, Schutte AE, Huisman HW, Rooyen JMv, Schutte R, Fourie CMT, Malan NT, Seedat YK. Conventional and behavioral risk factors explain differences in sub-clinical vascular disease between black and Caucasian South Africans: The SABPA study. Atherosclerosis. 2011; **215**: 237-42.
12. Doyon A, Kracht D, Bayazit AK, Deveci M, Duzova A, Krmar RT, Litwin M, Niemirska A, Oguz B, Schmidt BM, Sozeri B, Querfeld U, Melk A, Schaefer F, Wuhl E. Carotid artery intima-media thickness and distensibility in children and adolescents: reference values and role of body dimensions. Hypertension. 2013; **62**: 550-6.
13. Finken MJJ, Inderson A, Van Montfoort N, Keijzer-Veen MG, Van Weert AWM, Carfil N, Frolich M, Hille ETM, Romijn JA, Dekker FW, Wit JM, Study DP-C. Lipid profile and carotid intima-media thickness in a prospective cohort of very preterm

subjects at age 19 years: Effects of early growth and current body composition. Pediatric Research. 2006; **59**: 604-9.

14. Song YM, Lee K, Sung J, Kim YS, Lee JY. Sex-specific relationships between adiposity and anthropometric measures and carotid intima-media thickness in Koreans: The Healthy Twin Study. European Journal of Clinical Nutrition. 2012; **66**: 39-46.
15. Kim ES, Park J-H, Lee MK, Lee DH, Kang ES, Lee HC, Jekal Y, Jeon JY. Associations between Fatness, Fitness, IGF and IMT among Obese Korean Male Adolescents. Diabetes and Metabolism Journal. 2011; **35**: 610-8.
16. Moreno M, Puig J, Moreno-Navarrete JM, Xifra G, Ortega F, Ricart W, Fernandez-Real JM. Lean mass, and not fat mass, is an independent determinant of carotid intima media thickness in obese subjects. Atherosclerosis. 2015; **243**: 493-8.
17. Jourdan C, Wuhl E, Litwin M, Fahr K, Trelewicz J, Jobs K, Schenk JP, Grenda R, Mehls O, Troger J, Schaefer F. Normative values for intima-media thickness and distensibility of large arteries in healthy adolescents. Journal of Hypertension. 2005; **23**: 1707-15.
18. Millwood IY, Li L, Smith M, Guo Y, Yang L, Bian Z, Lewington S, Whitlock G, Sherliker P, Collins R, Chen J, Peto R, Wang H, Xu J, He J, Yu M, Liu H, Chen Z. Alcohol consumption in 0.5 million people from 10 diverse regions of China: prevalence, patterns and socio-demographic and health-related correlates. Int J Epidemiol. 2013; **42**: 816-27.
19. Du HD, Bennett D, Li LM, Whitlock G, Guo Y, Collins R, Chen JS, Bian Z, Hong LS, Feng SX, Chen XF, Chen LL, Zhou RX, Mao EK, Peto R, Chen ZM. Physical activity and sedentary leisure time and their associations with BMI, waist

circumference, and percentage body fat in 0.5 million adults: the China Kadoorie Biobank study. American Journal of Clinical Nutrition. 2013; **97**: 487-96.

20. Chen Z, Smith M, Du H, Guo Y, Clarke R, Zheng B, Collins R, Chen JS, Qian YJ, Wang XP, Chen XF, Tian XC, Wang XH, Peto R, Li LM, Collaboration CKB. Blood pressure in relation to general and central adiposity among 500 000 adult Chinese men and women. Int J Epidemiol. 2015; **44**: 1305-19.
21. Smith M, Li L, Augustyn M, Kurmi O, Chen J, Collins R, Guo Y, Han Y, Qin J, Xu G, Wang J, Bian Z, Zhou G, Peto R, Chen Z. Prevalence and correlates of airflow obstruction in approximately 317,000 never-smokers in China. Eur Respir J. 2014; **44**: 66-77.
